# Role of Visual Assessment of High-Quality Cakes in Emotional Response of Consumers †

**DOI:** 10.3390/foods11101412

**Published:** 2022-05-13

**Authors:** Jose Alba-Martínez, Andrea Bononad-Olmo, Marta Igual, Luís M. Cunha, Javier Martínez-Monzó, Purificación García-Segovia

**Affiliations:** 1 Food Investigation and Innovation Group, Food Technology Department, Universitat Politècnica de València, Camino de Vera s/n, 46022 Valencia, Spain; joalmar4@epsg.upv.es (J.A.-M.).; andrea.bononad@gmail.com (A.B.-O.); marigra@upvnet.upv.es (M.I.); xmartine@tal.upv.es (J.M.-M.); 2 GreenUPorto/Inov4Agro, DGAOT, Faculty of Sciences, Campus Agrário de Vairão, University of Porto, Rua da Agrária, 747, 4485-646 Vila do Conde, Portugal; lmcunha@fc.up.pt

**Keywords:** visual assessment, check-all-that-apply, emotions, EsSense Profile^®^

## Abstract

Thinking of the present gastronomic trends is inevitable when talking about innovation in haute pastry. Launching a successful product that meets consumers’ high expectations despite the rising demand for new creations is increasingly complex. For this reason, sensory analysis studies are more and more interested in studying the emotional response evoked by these products to better understand and improve user experiences. The main goal of this work was to conduct a study to analyze the emotional arousal of consumers after the visualization of five haute patisserie cakes. An online questionnaire with the EsSense Profile^®^ scale and CATA methodology were used for data collection. The EsSense Profile^®^ is a predefined and validated scale that measures food-related emotions, which includes 39 terms. When analyzing the emotions expressed by all the participants, 22 were statistically significant, of which 14 were classified as positive, 6 as neutral, and only 2 were negative. By analyzing the responses by gender, we observed differences in the number of elicited emotional terms: females showed significant differences between cakes for 18 emotion terms compared to 8 terms for males. The results obtained support the importance of the emotional profile to understand consumers’ expectations and behavior.

## 1. Introduction

The current concept of gastronomic innovation is experiencing growing public and professional interest [1]. Although culinary creativity has always existed, creativity focuses mainly on haute cuisine. Due to similar intuitive product development, this ‘modernist cuisine’ phenomenon has also affected traditional bakeries. Thus, its transformation into what is known as haute pastry has turned it into a technical, rigorous, and multidisciplinary area. These high-quality pastries could be considered an “ephemeral art”, which, in addition to technique, have an aesthetic and emotional sensitivity in the reconciliation of flavors, colors, textures, and shapes of the different ingredients that are part of each composition [2,3,4,5]. However, innovative creations give changes or modifications to recipes that can impact consumer perceptions and acceptance [6]. Four trends have been identified in pastry and bakery for the next years [7]: “eatertainment/innovative”, “fusion”, “indulgence”, and “identity/tradition”. The “eatertainment/innovative” trend is related to sophisticated designs and attractive appearances shared mainly on social media. “Fusion” concepts are inspired by global flavors, sweet-and-salty combinations, and unexpected textures. “Indulgence” is the joy that guilty pleasure brings, in which chocolate is the premium desired ingredient. “Identity/tradition” refers to a trend of a demand for comfort pastries.

Before tasting, the consumer creates a series of expectations based on intrinsic and extrinsic product cues. Intrinsic cues related to sensory characteristics (appearance, size, structure, texture, or flavor) determine food choice [8]. However, extrinsic characteristics (logo, brand, packaging, or retail context) contribute to generating these expectations [9]. The visual evaluation of these cues elicits a combination of physiological, emotional, and cognitive responses, often unconscious, playing a fundamental role in food choice and purchase decisions [10,11,12,13]. Consumers’ decisions to purchase food are based on visual cues provided by social media (Internet webpages, Instagram, Tik Tok, Twitter, YouTube, Twitch, etc.) [14]. The arrival of COVID-19 has accelerated the replacement of physical scenarios with virtual scenarios, from hospitals or universities to marketplaces or food suppliers [15]. Hence, to engage potential consumers, food companies need to pay attention to improving the visual impression of their products, considering intrinsic and extrinsic characteristics [16]. In commercial spaces, 80% of the information that the client receives is related to sight. Studying the role of senses in purchase decisions, the authors concluded that people remember 58% of information by sight, 45% by smell, 41% by hearing, 31% by taste, and 25% by touch [17,18]. In the past decade, studies in neuroscience have demonstrated how powerful a cue the sight of appealing food can be for the brain [19]. The visual exposure to appetizing food pictures of food not only activates regions of the visual cortex but also activates tasting circuits to produce conceptual inferences about taste [20].

A study to understand what food-related motives influenced consumers’ buying behaviors suggested that people prioritized emotional versus primary needs [21]. Therefore, visually speaking, the consumer is exposed to various emotional experiences, often unconsciously, when it comes to perceiving a high-quality pastry cake. When evaluating consumers’ emotional responses to food products, distinguished information contributes to understanding consumer behavior and expectations and the acceptance of new products [22]. These emotional responses that arise from the visual signals caused by the perception of a relevant stimulus [23] play an essential role in adding value to the product and directly controlling the expectations and perception of the consumer [24]. Indeed, consumers’ desire to purchase is very often driven by the unconscious emotional evocation of a product [25].

Empirical evidence has confirmed a stronger association between certain foods and gender stereotypes [26,27,28,29,30]. These studies suggested that foods may have gender connotations and play an essential role in food choice [31,32] across two dimensions: type and quantity [33]. Aspects such as healthy perception, portion size, dish presentation, or willingness to eat have been studied to relate to gender stereotypes [27,28,32,34]. Gender differences tend to be found in expressiveness but not in experience [35,36]. Women and men have the same emotion in the same situation (e.g., sadness when both are sad); however, women report being more emotionally expressive of most emotions than men [37].

Some specific questionnaires have been developed in different languages and cultures in the last decade to measure emotional responses to food [38]. The EsSense Profile^®^ can be considered the most referenced and validated [39,40,41]. Despite restrictions or difficulties in expressing emotions in diverse cultures or languages, this questionnaire based on 39 emotional terms remains helpful in the product development context, at least when a product name, the aroma of the product, or the flavor of the product were tested [41]. To our knowledge, no study has compared similarities or differences in food-elicited emotions between genders when the simple verbal terms of EsSense Profile^®^ are used, apart from the results shown by King et al., 2021 [40]. These authors evaluated the relationship between acceptability and emotions for different products and product categories. In their conclusion, they observed that emotions provide another way to look at gender differences in the products beyond acceptability scores.

The goals of this work were (i) to study consumers’ emotional response after visual evaluation of images of five specially designed cakes following market trends, (ii) to identify consumer emotions to be potentially used to evaluate willingness to buy and visual acceptability of these products, and (iii) to determine the influence of gender on cake-elicited emotions.

## 2. Materials and Methods

### 2.1. Stimuli

**Figure 1:**
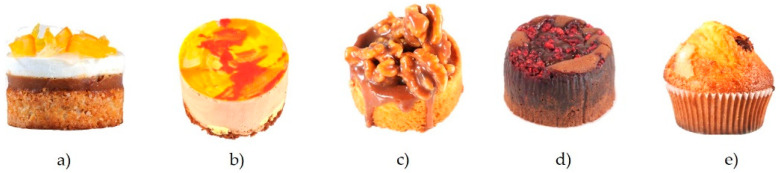
Stimuli designed by “Casa La Curra”: (**a**) Leonor cake (LC); (**b**) Saffron cake (SC); (**c**) Walnut cake (WC); (**d**) Coulant cake (CC); (**e**) Chocomuffin (CM).

Following the current pastry trends [42], five cakes were designed by “Casa La Curra” (Torrent, Valencia, Spain) to be used in this study (Figure 1). “Leonor cake” (LC) was made of an almond base and lemon mousse and decorated with candied orange pieces (Figure 1a). “Saffron cake” (SC) consisted of a base of butter biscuits filled with saffron cream and a chocolate mousse (Figure 1b). “Walnut cake” (WC) was made of a sablée breton with salted caramel cream and nuts (Figure 1c). A “Coulant cake” (CC) with chocolate and dried raspberries (Figure 1d) was created. Finally, the “Chocomuffin” (CM) is a traditional muffin filled with chocolate (Figure 1e).

Pictures were taken with a Nikon D300 camera with Nikkor 24–70 mm f/2.8S objective (Nikon Corporation, Tokyo, Japan). A white uniform background and three led panel light Neewer (Shenzhen Neewer Technology Co., Shenzhen, China) were used to ensure constant lighting conditions.

### 2.2. Participants Recruiting

Participants were invited to complete the online survey via email and social networks. The contact list included students, staff, lecturers of the Universitat Politècnica de Valencia, and other contacts. It is crucial to notice that, as is usual in qualitative research, this study did not intend to represent the entire Spanish population but rather to test the results of online questionnaires to obtain inferences about the emotional response to visual evaluation. A total of 381 answers were obtained and analyzed.

This study was reviewed and approved by The Institutional Review Board. Before engagement in the study, all participants gave informed consent. Data were treated anonymously following the European General Data Protection Regulation (Regulation E. C., 2016), assigning a random three-digit code to each participant.

### 2.3. Questionnaire

A specific online questionnaire was designed with the RedJade^®^ Online Survey Tool (Redjade Sensory Solutions, LLC, Martinez, CA, USA). The questionnaire included three parts: (i) a brief food frequency questionnaire of sweet products and subjective hunger, (ii) visual evaluation using the EsSense Profile^®^ (39 emotions) [43] in a randomized CATA list, and (iii) sociodemographic questions. The time to answer was not longer than 10 min. The online questionnaire was available for 2 weeks.

### 2.4. Statistical Analysis

For sociodemographic data, basic descriptive statistics and variable characterization were carried out. Visual acceptance scores were analyzed using analysis of variance (ANOVA), taking “gender”, “age”, “level of education”, “nationality”, and “cakes” as fixed effects and “participant” as a random effect. Only significant variables were presented in the final model.

Cochran’s Q test was performed to determine which emotions discriminated between samples (*p* < 0.05). Correspondence analysis (CA) was used to investigate the association between the cakes and the expected emotions. Principal component analysis (PCA) was performed with the emotions and several variables: visual acceptance, willingness to buy, and gender. According to Tukey’s test, multiple mean comparisons were carried out to determine significantly different groups.

Cluster analysis was applied to classify the participants into homogeneous classes according to evoked emotions by cake.

A multiple factor analysis (MFA) was performed on CATA counts to determine the effect of gender on emotional sample evaluation [44,45]. The regression vector (RV) coefficient was calculated between the first three axes of the partial configurations from MFA to analyze the similarity between sample evaluations from both genders.

All analyses were performed using XLSTAT statistical software version 2021 [46].

## 3. Results and Discussion

### 3.1. Participants’ Profile and Visual Acceptance

The profile of the 381 participants included in this study were 70% women and 29% men. Participants were mainly Spanish (91%), with 5% from Europe, 3% from Latin America, and the remaining 1% corresponded to Africa, China, and the United States. The age ranged from 14 to 77 years old, with an average of 42 ± 15 years old. Bachelor’s degree was the most frequent level of study among the participants in general (35%). In particular, 23% of young people (from 14 to 30 years old) held a bachelor’s degree, and 28% of adults (from 31 to 77 years old) declared a PhD degree.

Results for differences in participants’ visual acceptance of the five cakes analyzed with ANOVA mixed models are presented in Table 1. Only the factors cake and the interaction cake*age (fixed) and participant (random) provided significant information to explain the variability in the visual acceptance. Other sociodemographic factors such as the origin of participants, level of hunger, or gender did not bring meaningful information to explain the variability of visual acceptability and were removed from the final model.

**Figure 2:**
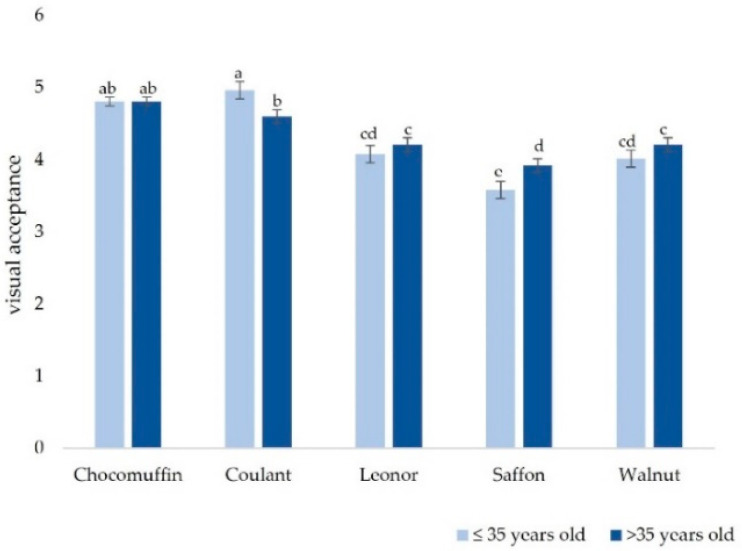
Average visual acceptability classified by cake and age group. The different lowercase letters on the columns indicate the significantly different groups according to Tukey’s test.

As shown in Figure 2, the visual acceptability varied by “cake” and by “age”. There were no significant differences in ages for Chocomuffin and Coulant, which were well-known and traditional cakes. No differences between Leonor and Walnut cakes were found, although they had a lower score in visual acceptability. Saffron cake had a lower acceptability compared with other cakes, and significant differences in ages were found.

### 3.2. Emotional Profile after Visual Evaluation

The CATA data analysis was initially conducted with 39 emotions used in the EsSense Profile^®^. Cochran’s Q test (Table 2) allowed to choose only those that showed significant differences between the different cakes (*p* < 0.05). Consequently, 22 significant emotions were selected from the EsSense Profile^®^ list, with which the emotional profile of each of the five cakes was studied (Table 1). From this list, 14 are positive, 6 are neutral, and 2 are negative emotions.

**Figure 3:**
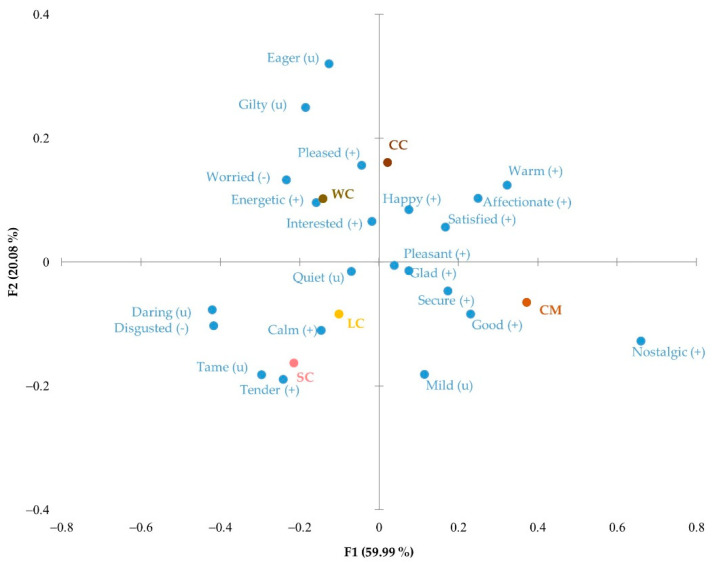
Correspondence analysis of the significant emotional responses obtained with CATA EsSense Profile™: (+), positive emotion; (−), negative emotion; (u), neutral emotion; WC, Walnut cake; LC, Leonor cake; SC, Saffron cake; CC, Coulant cake; CM, Chocomuffin.

Figure 3 shows the correspondence analysis of the significant emotions. The more innovative Leonor (LC) and Saffron (SC) cakes were associated with “calm” and “tender” positive emotions and with neutral emotions such as “tame” and “quiet”. On the other hand, Chocomuffin (CM) was linked to the emotions “secure”, “good”, and “nostalgic” because of its widespread and frequent consumption, which was associated with positive emotions that bring back good memories. Walnut (WC) and Coulant cakes (CC) were relatively close on the emotional map, although Coulant cake was more related to “happy” and “pleased”, perhaps because it was made of chocolate, an appreciated comfort ingredient commonly associated with pleasure [7]. Walnut cake made participants feel “energetic” and “worried” because of its attractiveness but caloric ingredients generated a feeling of concern, as it was also perceived as a source of energy.

### 3.3. Visual Acceptance and Willingness to Buy

**Figure 4:**
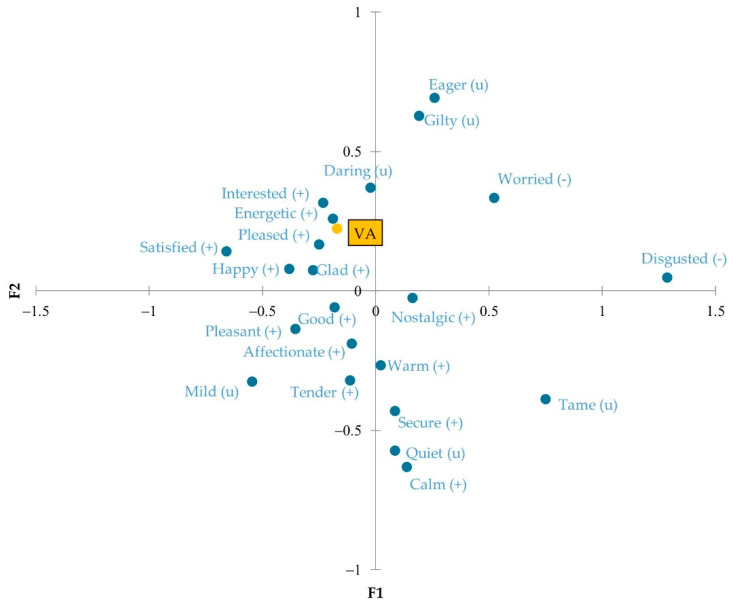
Principal coordinates analysis with the variable “visual acceptance” (VA) of the significant emotional responses obtained with CATA EsSense Profile^®^: (+), positive emotion; (−), negative emotion; (u), neutral emotion.

The variables “visual acceptance” (VA) and “willingness to buy” (WTB) were analyzed to identify the emotions to which they are related. Figure 4 represents the principal coordinate analysis in the form of an emotional map that places VA among the different significant attributes. It can be said that a cake with greater visual acceptance is mainly associated with the emotions “energetic” and “interested”. On the other hand, the one mainly related to “disgusted” produces a certain visual rejection. Again, it is striking that when a cake is related to the neutral adjectives “daring” and “guilty”, more visual tolerance is generated than with the adjectives “secure” and “calm”, classified as positive. These emotions, related to trust, are common when evaluating a widely accepted product [47,48]. Therefore, given the importance of visual acceptance in consumer expectations, this analysis shows important aspects to be considered.

**Figure 5:**
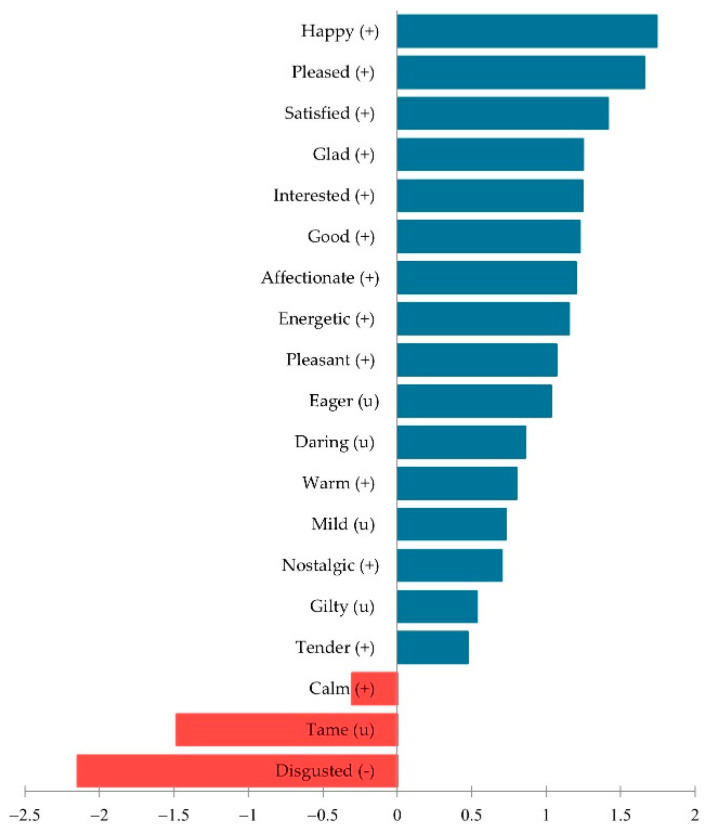
Mean impact in willingness to buy for each significant emotion on CATA analysis: (+), positive emotion; (−), negative emotion; (u), neutral emotion.

Figure 5 shows the penalty analysis used to identify the emotions that led to a lower, no effect, or a higher effect on the willingness to buy mean. As can be observed, only three emotions (“calm”, “disgusted”, and “tame”) had a negative effect. It is suggestive that the adjective “disgusted” is the most common when the respondent evaluates a product that is considered unpleasant [42,43]. On the other hand, “happy”, “pleased”, and “satisfied” are the ones that have positive effects, and “secure”, “quiet”, and “worried” did not show an effect on the willingness to buy mean. This analysis provides insights on which emotions must get evoked and which must not be induced when designing a new cake to be successful in the market, based on participants’ profiles.

**Figure 6:**
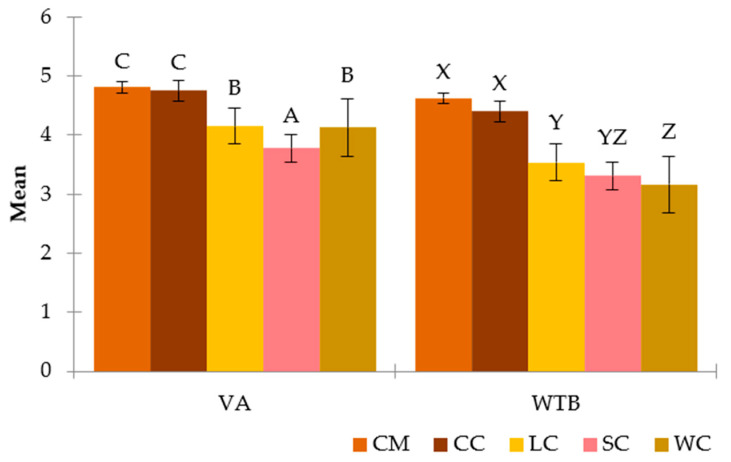
Multiple mean comparisons according to the visual acceptance (VA) and willingness to buy (WTB) for each cake. The different capital letters on columns indicate the significantly different groups according to Tukey’s test: WC, Walnut cake; LC, Leonor cake; SC, Saffron cake; CC, Coulant cake; CM, Chocomuffin.

Tukey’s HSD multiple comparison test was performed to compare each group of cakes according to the variables VA and WTB (Figure 6). Saffron cake has a significantly lower value of VA than the rest, which makes it the least visually appreciated by the participants. Leonor and Walnut cakes presented significant differences with Saffron cake, Chocomuffin, and Coulant cake. Moreover, the last group without significant differences is Chocomuffin and Coulant cake, the most visually accepted. These latter two cakes are the best known and most familiar to the respondents, so the fact of being used to them directly influences their general acceptance in a positive way [47].

As for the WTB variable, the results did not follow the same distribution. The two cakes that inspired the most desire to buy were Chocomuffin and Coulant cake, with a slight nonsignificant preference over the first one. No statistically significant differences were found in the WTB of Leonor and Saffron cakes, which did appear with Chocomuffin and Coulant cake, and only Leonor cake presented differences with Walnut cake. Finally, Walnut and Saffron cakes form a homogeneous group with statistical differences from the one formed by Chocomuffin and Coulant cakes. It should be noted that despite being the worst valued visually, Saffron cake shows a greater willingness to buy than Walnut cake, which had a greater VA.

### 3.4. Cluster Analysis

Once relating the attributes to each cake, a cluster analysis was carried out to classify the participants into homogeneous groups according to CATA. Three differentiated classes are obtained when clustering the answers. A different correspondence analysis (CA) was obtained from each class, which allows for distinguishing the emotional responses for each cake. In the three classes, the CA’s first axis separated cakes into the same two groups: innovative (Saffron, Leonor, and Walnut cake) and well-known (Coulant and Chocomuffin).

**Figure 7:**
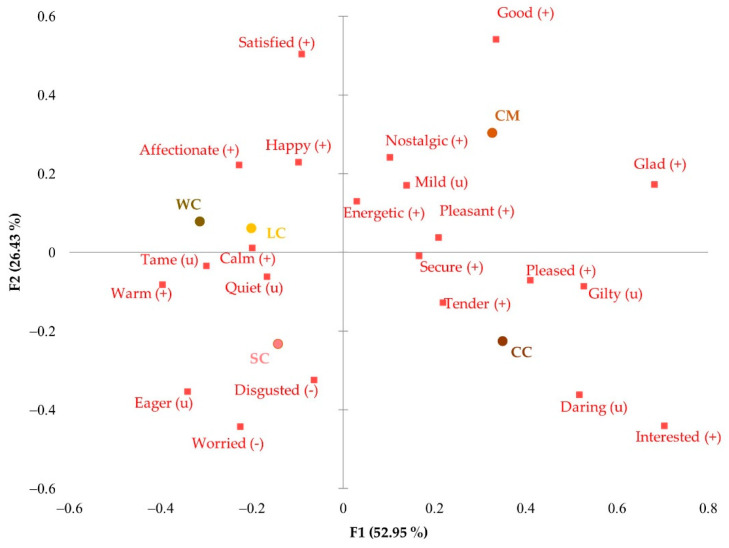
Principal component analysis of the significant emotional responses in class 1: WC, Walnut cake; LC, Leonor cake; SC, Saffron cake; CC, Coulant cake; CM, Chocomuffin.

Class 1 was composed of 69 participants. As shown in Figure 7, Coulant and Chocomuffin were associated with positive or neutral emotions but with positive effects on the visual acceptability mean when talking about sweet cravings. The differentiation was between “good” and “glad” for the Chocomuffin and “guilty”, “interested”, and “daring” for the Coulant cake. The Saffron cake was closely related to the negative emotions of “disgusted” and “worried”. In the case of the Leonor and Walnut cakes, “quiet” and “calm” were the emotions closely associated.

**Figure 8:**
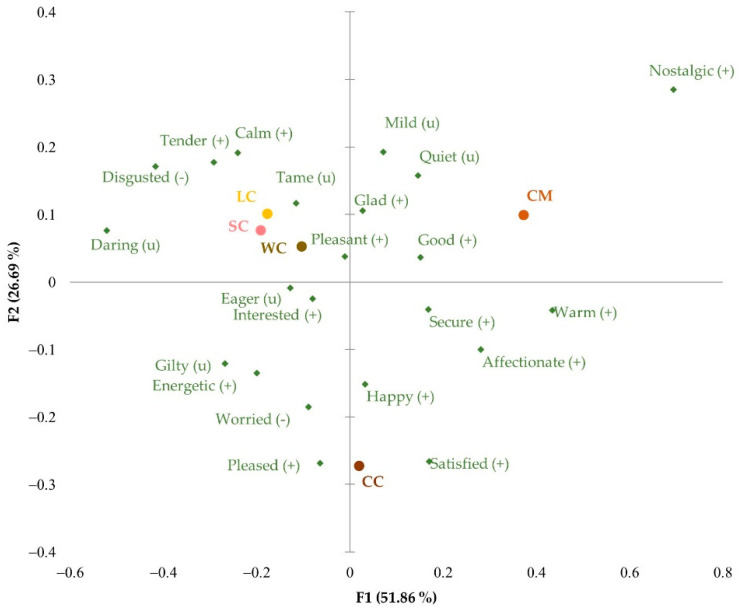
Principal component analysis of the significant emotional responses corresponding to class 2: WC, Walnut cake; LC, Leonor cake; SC, Saffron cake; CC, Coulant cake; CM, Chocomuffin.

Class 2 encompassed the largest number of participants—210 people— who presented different emotional profiles (Figure 8). The emotions “disgusted”, “tame”, and “calm”, with a negative impact on willingness to buy (Figure 5), were closer to the more innovative cakes (Leonor, Saffron, and Walnut cakes). This may indicate that the members in class 2 were not particularly attracted to the unknown cakes or may even dislike them, hinting at being more traditional in their choices. Chocomuffin was associated with positive feelings, highlighting “nostalgic” and “good”, reflecting the participants’ approval. The visualization of the Coulant cake evoked feelings of pleasure, happiness, and satisfaction, as it is a classic and habitual product that can trigger a craving.

**Figure 9:**
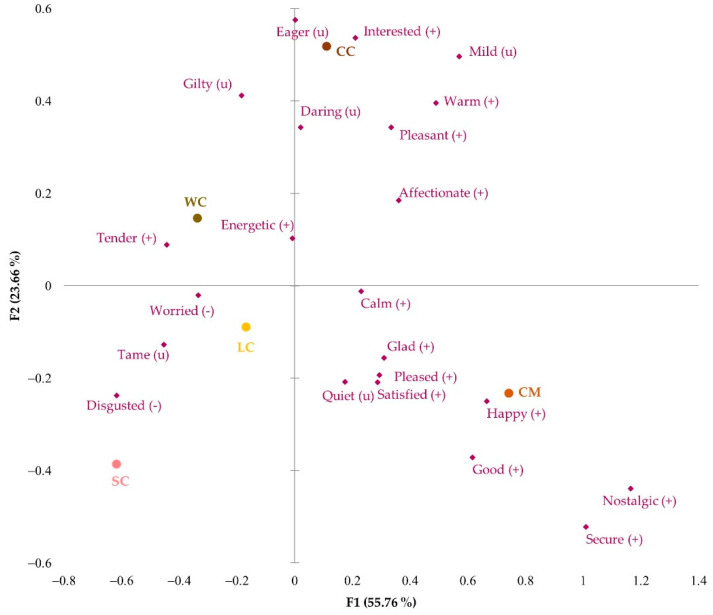
Principal component analysis of the significant emotional responses corresponding to class 3: WC, Walnut cake; LC, Leonor cake; SC, Saffron cake; CC, Coulant cake; CM, Chocomuffin.

In contrast, class 3 (*n* = 100) presented a different profile (Figure 9). The first axis of the CA also included the two most well-known and traditional cakes (Chocomuffin and Coulant); however, they had an opposite position on the second axis. Coulant cake was surrounded by neutral emotions: “eager”, “mild”, “daring”, and “guilty”, and closely related with “interested” as a positive emotion. Saffron and Leonor cakes evoked, in this class, the neutral emotions “disgusted”, “worried”, and “tame”, though with a negative effect. The most positive emotions, “nostalgic”, “happy”, “secure”, and “good”, were correlated with the Chocomuffin.

### 3.5. Emotional Profile by Gender

According to the participants’ gender, emotional responses were studied to see the changes in the significant emotions and their effect on each cake. Cochran’s Q test (Table 3) was used to determine if gender affected emotional profile regarding differences between cakes. The emotions “happy”, “affectionate”, ” pleasant”, “satisfied”, and “tender” were significant for women but not for men.

The cake-by-cake analysis revealed that, in general, women were more descriptive in the emotions evoked. In the Cochran’s Q test, 19 of the 22 general emotions selected were significant in the analysis of the emotional profile of women. In the case of men, nine attributes showed significant differences, according to the Cochran’s Q test. According to previous studies, the gender differences were in emotional expressiveness but not in emotional experience [32,33,34]. The emotional profile signaled by women showed more expressiveness than men. These data agree with King et al.’s 2010 results, which evaluated the relationship between acceptability and emotions for different products and product categories and found that, on average, females rate emotion intensities stronger than do males [40].

A Fisher exact test was performed to compare differences in frequency of each of the 22 sensory terms by gender. Only results for the emotion “tame” were significant (Table 4).

**Figure 10:**
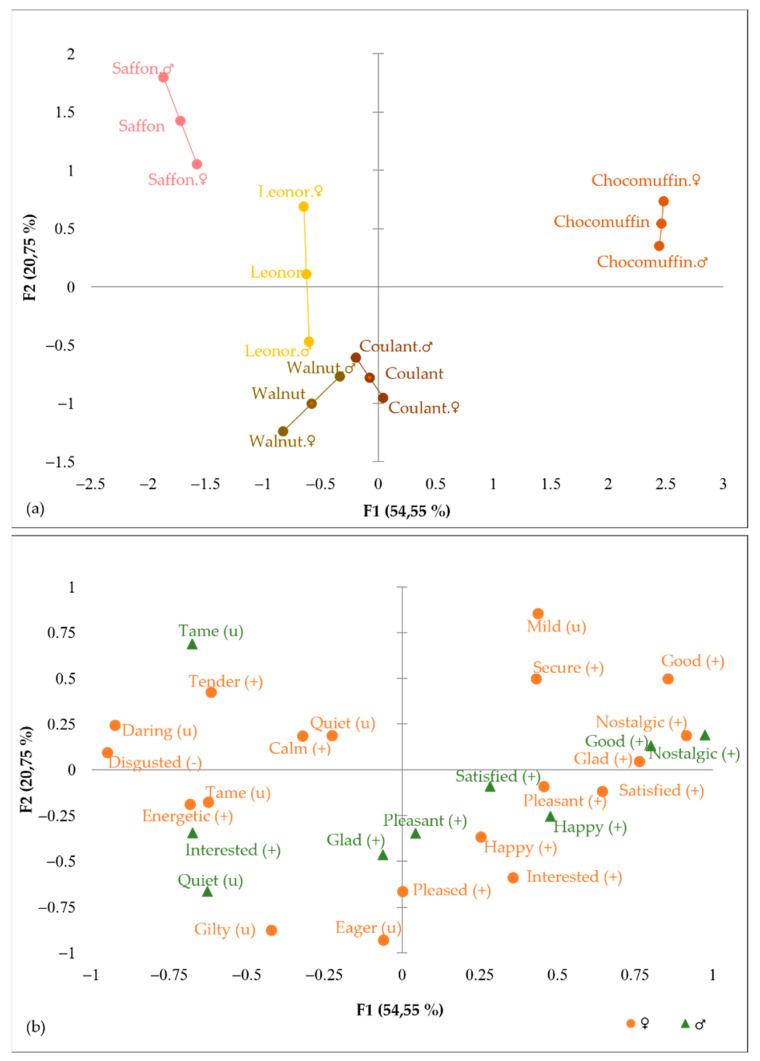
MFA on CATA emotions by gender: (**a**) cakes representation; (**b**) emotion terms of CATA EsSense Profile^®^.

Multiple factor analysis (MFA) was carried out to compare the emotional profile obtained from CATA terms of both genders. The RV coefficient between cakes was 0.884, indicating that the emotional profile derived from the two genders was similar but not identical. The position of the cakes on the product map was affected by gender interaction. Figure 10a shows that the difference between cakes according to gender was more considerable for Saffron and Leonor cakes than Coulant or Chocomuffin.

Figure 10b shows the projection of emotions by gender. There were some emotions highly correlated with gender, while others were poorly correlated. The emotion “nostalgic” was projected close together, indicating that this emotion was similarly described in the emotions evoked for the five cakes evaluated in both genders. Analysis of multiple pairwise comparisons indicated that the “nostalgic” emotion differed statistically in Chocomuffin from other cakes in both genders.

On the opposite side, emotions such as “glad” or “interested” were poorly correlated, suggesting a different use of these terms to describe the emotions evoked in both genders. In the multiple analysis comparison, the data for women and men indicated that the five cakes did not differ in the emotion “interested”, while the effect of the cake was significant between Saffron cake and Chocomuffin for the “glad” emotion in the women’s evaluation.

## 4. Limitations

These results should be observed with caution due to some limitations of this study. First, the study used mainly Spanish participants (91%) with a high level of education (83% held a higher degree), and they were aged between the Boomers and Millennials. A more diverse population should be represented in the following steps to generalize these results. On the other hand, only the impact of appearance (visual stimulus) on the emotional response was studied in this work. To obtain the complete emotional response evoked by the overall sensory stimuli, smell and taste experiences will need to be incorporated into further research. To understand gender differences in future research, an intensity scaled approach in emotional terms must be used to obtain more detail, specifically when small product differences are compared.

## 5. Conclusions

The preliminary results of this work support the importance of the emotional profile for understanding consumers’ emotions in the visual evaluation of cakes. In the case of haute pastry, visual appearance had an impact on the participants, evoking several emotions. However, different results may perhaps be achieved if other sensory properties (smell and taste) were evaluated. Gender differences were found in expressiveness (number of emotions elicited) but not in experience (the same emotion by the same cake). This work opens the authors to future areas of study to understand the effects of emotions on eating behavior for haute pastry products, including the perspective of gender.

## Figures and Tables

**Table 1:** ANOVA mixed models to test significant differences between fixed and random effects.

Source	Type	DF	Sum of Squares	F	Pr > F
Participant	Random	379.000	2271.176	4.171	<0.0001
Cake	Fixed	4.000	315.572	54.909	<0.0001
Age group × Cake	Fixed	4.000	26.292	4.575	0.001
Error		1516.000	2178.170		

**Table 2:** Cochran’s Q test for the comparison of emotion elicitation by cake type for each emotion term.

Emotion	*p*-Values	Emotion	*p*-Values	Emotion	*p*-Values
Bored (−)	0.420	**Happy (+)**	**<0.0001**	**Good (+)**	**<0.0001**
Joyful (+)	0.281	**Nostalgic (+)**	**<0.0001**	**Guilty (u)**	**<0.0001**
**Daring (u)**	**0.000**	**Mild (u)**	**0.004**	**Energetic (+)**	**0.011**
**Warm (+)**	**0.000**	**Affectionate (+)**	**0.008**	Whole (+)	0.057
Wild (u)	0.071	Loving (+)	0.466	**Worried (−)**	**0.009**
Steady (u)	0.214	Good nature (+)	0.310	**Disgusted (−)**	**<0.0001**
Free (+)	0.753	**Glad (+)**	**0.008**	**Calm (+)**	**0.014**
**Secure (+)**	**0.007**	**Pleased (+)**	**0.001**	**Tender (+)**	**0.003**
Active (+)	0.690	**Tame (u)**	**0.002**	Enthusiastic (+)	0.054
Friendly (+)	0.127	Peaceful (+)	0.108	**Interested (+)**	**0.028**
Adventurous (+)	0.765	**Quiet (u)**	**0.002**	**Satisfied (+)**	**<0.0001**
Understanding (u)	0.617	**Pleasant (+)**	**0.018**	Merry (+)	0.271
Polite (u)	0.883	**Eager (u)**	**<0.0001**	Aggressive (u)	0.066

Terms with significant differences (*p* < 0.05) are shown in bold: (+), positive emotion; (−), negative emotion; (u), neutral emotion.

**Table 3:** Cochran’s Q test for significant differences between the 22 selected emotion terms of the CATA EsSense Profile^®^ by gender.

	*p*-Value			*p*-Value	
Emotions	♀	♂	Emotions	♀	♂
Daring (u)	**0.001**	**0.031**	**Pleasant (+)**	**0.023**	0.459
Warm (+)	**0.032**	**0.032**	**Eager (u)**	**0.000**	0.059
**Secure (+)**	0.160	**0.030**	Good (+)	**<0.0001**	**0.002**
**Happy (+)**	**<0.0001**	0.322	Guilty (u)	**0.000**	**0.022**
Nostalgic (+)	**<0.0001**	**<0.0001**	Energetic (+)	0.050	0.303
**Mild (u)**	**0.043**	0.146	**Worried (−)**	**0.005**	0.371
**Affectionate (+)**	**0.018**	0.474	**Disgusted (−)**	**<0.0001**	0.395
**Glad (+)**	**0.014**	0.093	Calm (+)	0.082	0.106
**Pleased (+)**	**0.004**	0.082	**Tender (+)**	**0.004**	0.234
Tame (u)	**0.030**	**<0.0001**	Interested (+)	0.111	0.098
Quiet (u)	**0.031**	**0.036**	**Satisfied (+)**	**<0.0001**	0.566

Terms with significant differences (*p* < 0.05) between cakes are shown in bold for each gender.

**Table 4:** Frequency of the emotions of the CATA EsSense Profile^®^ by gender and results from Fisher’s exact test.

Emotions	♀	♂	*p*-Value	Emotions	♀	♂	*p*-Value
Daring (u)	81	43	0.323	Pleasant (+)	276	109	0.325
Warm (+)	63	30	0.733	Eager (u)	86	25	0.075
Secure (+)	91	47	0.398	Good (+)	204	97	0.517
Happy (+)	212	89	0.746	Guilty (u)	106	50	0.659
Nostalgic (+)	76	37	0.606	Energetic (+)	114	62	0.181
Mild (u)	164	69	0.770	Worried (−)	50	21	0.898
Affectionate (+)	50	29	0.267	Disgusted (−)	91	35	0.556
Glad (+)	220	78	0.091	Calm (+)	154	66	0.881
Pleased (+)	139	49	0.195	Tender (+)	114	53	0.732
**Tame (u)**	94	66	**0.004**	Interested (+)	171	80	0.672
Quiet (u)	255	95	0.164	Satisfied (+)	182	87	0.538

Term with significant differences (*p* < 0.05) is shown in bold.

## Data Availability

Data is contained within the article.
